# Negative Predictive Value of Human Papillomavirus Testing: Implications for Anal Cancer Screening in People Living with HIV/AIDS

**DOI:** 10.1155/2020/6352315

**Published:** 2020-01-22

**Authors:** Yiying Wang, Yan Wang, Michael M. Gaisa, Keith Sigel, Wenxin Zheng, Yuxin Liu, Yue Wang

**Affiliations:** ^1^Department of Obstetrics and Gynecology, Henan Provincial People's Hospital, Zhengzhou University People's Hospital, Henan University People's Hospital, Zhengzhou, Henan 450003, China; ^2^Department of Pathology, University of Texas Southwestern Medical Center, Dallas, TX, USA; ^3^Department of Medicine, Division of Infectious Disease, Icahn School of Medicine at Mount Sinai, New York, NY, USA; ^4^Department of Medicine, Division of General Medicine, Icahn School of Medicine at Mount Sinai, New York, NY, USA; ^5^Department of Obstetrics and Gynecology, Simons Comprehensive Cancer Center, University of Texas Southwestern Medical Center, Dallas, TX, USA; ^6^Department of Pathology, Icahn School of Medicine at Mount Sinai, New York, NY, USA

## Abstract

**Objectives:**

People living with HIV/AIDS (PLWHA) have an increased incidence of anal squamous cell carcinoma. Since high-risk human papillomavirus (hrHPV) is the primary cause, hrHPV DNA testing may play an important role in anal cancer screening. This study aims to determine the negative predictive value (NPV) of hrHPV testing in PLWHA as well as factors that may lead to false-negative results.

**Methods:**

Anal swabs were collected for cytology and Cobas® 4800 HPV test for 14 hrHPV types. Patients underwent concomitant high-resolution anoscopy (HRA) examination and biopsy. High-grade squamous intraepithelial lesions (HSIL, synonymous with anal intraepithelial neoplasia AIN2 and 3) detected in Cobas-negative patients were genotyped for 22 HPV types using BioPerfectus Multiplex Real-time PCR.

**Results:**

156 PLWHA tested negative for hrHPV on anal swab samples (i.e., Cobas-negative). HRA-guided biopsy detected HSIL/AIN3 in 13 patients (8%, NPV 92%), HSIL/AIN2 in 5 patients (3%), low-grade squamous intraepithelial lesions in 82 (LSIL, 53%), or benign findings in 56 (36%). No cancer was found. The HSIL group was similar to the LSIL/benign group regarding age, gender, race/ethnicity, clinical HIV parameters, cytological diagnoses, history of receptive anal sex, and smoking (*p* ≥ 0.02). Genotyping HSIL tissue derived from Cobas-negative patients revealed hrHPV (*n*=7), possibly carcinogenic HPV53, 67, 73, 82 (*n*=12), or absence of hrHPV (*n*=4).

**Conclusions:**

In this series, anal hrHPV DNA testing offered 92% NPV for PLWHA; in other words, a 8% risk of occult precancer remains for those who test hrHPV negative on anal swab samples.

## 1. Introduction

Human papillomavirus- (HPV) associated anal cancer has been on the rise with a projected incidence of 8,300 new cases and 1,280 deaths in the United States in 2019 [1]. Due to the strong synergistic relationship between HPV and human immunodeficiency virus (HIV), people living with HIV/AIDS (PLWHA) have a significantly higher incidence compared to the general population, even for those receiving effective antiretroviral therapy [2, 3]. The highest anal cancer incidence is found in HIV-infected men who have sex with men (MSM), ranging from 77 to 137 per 100,000 [4, 5]. Accordingly, the HIV Medicine Association of the Infectious Diseases Society of America (IDSA) recommends anal cancer screening for PLWHA in order to detect and manage high-grade squamous intraepithelial lesions (HSIL, synonymous with anal intraepithelial neoplasia AIN2 and 3), the immediate precursors to anal cancer [6]. Since most patients with anal HSIL are asymptomatic or present with nonspecific symptoms, using screening modalities with high positive and negative predictive values becomes all the more important.

In the case of cervical cancer screening, both exfoliative cytology and high-risk HPV (hrHPV) testing have proven largely successful [7]. Given the significant overlap between cervical and anal HPV carcinogenesis, it is reasonable to expect that both methods will be effective in anal cancer screening [8]. However, as a result of the high prevalence of HPV-associated lesions and spectrum of different HPV types reported among HIV-infected MSM, anal cytology and hrHPV testing both demonstrate high sensitivity (81% and 95%, respectively) but low specificity (53% and 24%) [9]. Anal cytology is further hampered by substantial interobserver variability among cytopathologists, underscoring its deficiency as a sole screening test for anal cancer [10].

By contrast, recent research has demonstrated the important prognostic value of oncogenic HPV in anal carcinogenesis, particularly HPV16, reinforcing the role of hrHPV testing in anal cancer screening [11]. Studies have shown that HPV16/18 genotyping predicts the presence of anal HSIL, improves screening specificity as well as positive predictive value, and even provides long-term risk stratification for anal precancer [12, 13].

Negative predictive value (NPV) is an important performance measure for screening tests, especially those used in populations with high disease prevalence [14]. Tests with high NPV provide the clinical confidence of ruling out particular conditions, thereby avoiding unnecessary referrals and procedures. To achieve optimal use of hrHPV testing in anal cancer screening, it is critical to determine its NPV as well as factors that may lead to false negative results. Herein, we used concomitant biopsy results of HSIL/AIN3 as an endpoint to evaluate the NPV of hrHPV testing in a large cohort of PLWHA. Furthermore, we sought to provide insight into false negative cases defined as anal HSIL detected in the setting of Cobas-negative hrHPV.

## 2. Methods

### 2.1. Patient Selection and Demographics

The Mount Sinai anal dysplasia program, part of a large urban HIV clinic system, serves as a referral center for the diagnosis and treatment of PLWHA with HPV-associated anal precancer and cancer. According to current practice guidelines endorsed by IDSA, PLWHA with risk factors for anal cancer is screened by primary care or infectious disease physicians using anorectal cytology. If anorectal cytology is abnormal, defined as Atypical Squamous Cells of Undetermined Significance (ASCUS) or worse, patients are referred to the anal dysplasia program for high-resolution anoscopy (HRA) examination and biopsy. At the time of HRA, we routinely repeat anorectal cytology and hrHPV testing.

After obtaining Icahn School of Medicine Institutional Review Board approval, we searched the HRA database from January 2015 to December 2018 for PLWHA who tested negative for hrHPV at the time of HRA and biopsy. The following demographics were collected from the electronic medical record: age, gender, race/ethnicity, sexual behavior, history of receptive anal sex, HIV status, CD4+ T-cell count, HIV-1 viral load, and smoking history.

### 2.2. Anorectal Cytology and HPV DNA Testing

Anal swabs were performed using a moistened cytobrush to survey the anal canal mucosa from the anal verge to right above the squamocolumnar junction. Samples were preserved in liquid-based cytology medium for ThinPrep® Pap Test (Cytyc Corp., Boxborough, MA) and stained with the Papanicolaou stain. Cytopathologists from the Mount Sinai Hospital reported all cases using the 2001 Bethesda System: unsatisfactory (≤two nucleated squamous cells/high-power field); benign; ASCUS; low-grade squamous intraepithelial lesion (LSIL); atypical squamous cells, cannot exclude HSIL (ASC-H); and HSIL [15]. Regardless of cytological diagnosis, an aliquot of the swab sample was tested for hrHPV DNA using the Cobas®4800 system (Roche Diagnostics, Indianapolis, IN) following manufacturer instructions. The assay reports HPV16, 18, and pooled results for 12 hrHPV types: 31, 33, 35, 39, 45, 51, 52, 56, 58, 59, 66, and 68.

### 2.3. HPV Genotyping on Biopsy Samples

Anal HSIL biopsy samples derived from Cobas-negative patients were genotyped using the BioPerfectus Multiplex Real Time (BMRT) PCR assay. DNA was extracted from biopsy samples using QIAamp DNA mini kit (Qiagen, Germany) according to the manufacturer's instructions. PCR was performed using the fluorescence-based multiplex HPV DNA genotyping kit (Bioperfectus Ltd., China) that is designed to identify 19 high-risk or possibly carcinogenic HPV types (16, 18, 26, 31, 33, 35, 39, 45, 51, 52, 53, 56, 58, 59, 66, 67, 68, 73, and 82) and 3 low-risk HPV types (6, 11, and 81) [16]. Results were analyzed using the Perfectus Software v1.0 (Bioperfectus Ltd., China).

### 2.4. HRA-Guided Biopsy and Histological Diagnosis

First, anal swabbing was performed to collect samples for cytology and Cobas HPV test. Subsequently, HRA and biopsies were performed following standard protocols [17]. After treatment with 3% acetic acid and Lugol's iodine, the perianal region, distal anal canal, and squamocolumnar junction were examined using a high-resolution colposcope at 15-fold magnification to look for abnormal vascular patterns and other signs of HSIL or cancer, including ulceration, mass effect, and mucosal friability. Biopsies were taken from areas suspicious for HSIL or cancer. Biopsy samples were processed following standard histological protocols. Surgical pathologists from the Mount Sinai Hospital reported all cases using the standard morphological criteria as outlined in the Lower Anogenital Squamous Terminology project [18]. The designation of HSIL required dysplastic cells with significant nuclear enlargement, coarse chromatin, and irregular nuclear membrane present in the middle third (Anal Intraepithelial Neoplasia 2, AIN 2) or top third of the epithelium (AIN 3).

The authors YL and WZ independently reviewed H&E slides for all cases and confirmed the diagnoses. Both authors are specialized gynecological pathologists with more than ten years experience in diagnosing HPV-associated anogenital disease. Only those cases with consensus diagnosis were included in the study. p16 immunohistochemistry (IHC) was performed on a subset of lesions. For small lesions, p16 IHC was not performed in order to preserve sufficient tissue for DNA isolation and HPV genotyping.

### 2.5. Statistical Analysis

Differences in patient demographics between subjects with benign findings vs. LSIL vs. HSIL were compared using the chi-square test or Fisher's exact test for categorical or binary variables as well as the Wilcoxon test for continuous variables (age, CD4+ T-cell count), as appropriate. All analyses were performed using STATA 15 (Stata Corporation, College Station, TX).

## 3. Results

**Figure 1:**
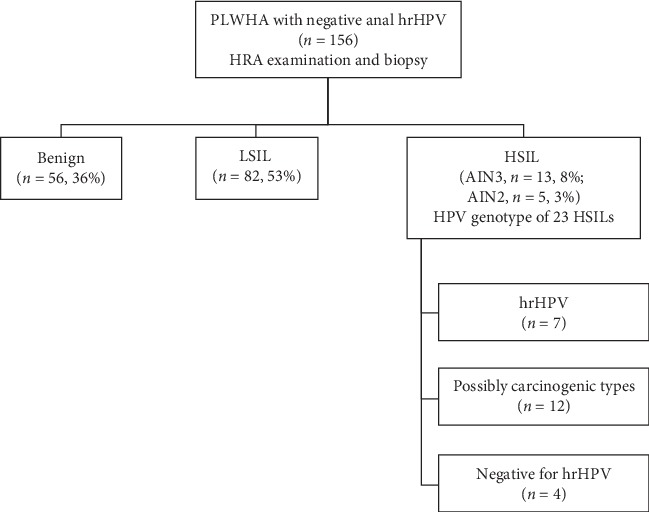
Study flow chart.

The study flow is shown in Figure 1. During the study period, 156 PLWHA tested negative for the 14 hrHPV types in the Cobas® panel on anal swab samples (i.e., Cobas-negative). Patient demographics are shown in Table 1. The median age was 49 years (range, 23–71). Most were MSM (83%) with a self-reported history of receptive anal sex (93%). All subjects were prescribed antiretroviral therapy. Most had CD4+ cell count ≥500 cells/mL and HIV-1 viral load <100 copies/mL (79% and 90%, respectively). Race and ethnicity included Hispanic (38%), Caucasian (26%), African American (22%), and others (14%). Thirty-five subjects (22%) were current smokers. At the time of HRA, repeated anorectal cytology was diagnosed as unsatisfactory (1%), benign (48%), ASCUS (40%), LSIL (8%), and ASC-H and HSIL (3%).

All Cobas-negative individuals underwent HRA examination and biopsy (median 4 biopsies per patient, range 2–9). Histological examination of biopsies revealed HSIL/AIN3 in 13 individuals (8%, NPV 92%), HSIL/AIN2 in 5 patients (3%), LSIL in 82 (53%), and benign findings in 56 (36%). In the HSIL group, 14 individuals had a solitary high-grade lesion, 3 had two high-grade lesions, and one individual had three high-grade lesions. In total, 23 biopsy-proven HSILs were identified including 13 AIN3 and 10 AIN2. Two experienced pathologists confirmed HSIL diagnosis for all cases through independent review. p16 IHC was performed on 8 lesions and revealed block-positive staining, supporting the HSIL diagnosis.

Demographics were compared between the benign, LSIL, and HSIL groups (Table 1). There was no statistically significant difference between the three groups, regarding age, gender, sexual behavior, receptive anal sex, clinical HIV parameters, race/ethnicity, smoking history, and cytology diagnoses (*p* ≥ 0.02).

The 23 HSILs derived from Cobas-negative patients were genotyped for HPV using BMRT assay. As shown in Table 2, most lesions (83%) harbored a single HPV type, while four lesions were coinfected by two HPV types. The most common types identified were HPV67 (*n*=5) and HPV82 (*n*=5). Seven lesions were positive for hrHPV types included in the Cobas® panel (31, 33, 35, 45, 51, 52, 58, and 59). Twelve lesions were positive for possibly carcinogenic HPV types 53, 67, 73, and 82 classified as the International Agency for Research on Cancer (IARC) category 2B and not included in the Cobas® panel [19]. Four lesions were negative for any of the 22 HPV types included in the BMRT assay.

## 4. Discussion

In this retrospective study, using biopsy-proven anal HSIL/AIN3 as an endpoint, we found that the hrHPV DNA test had a NPV of 92% for PLWHA undergoing anal cancer screening at our clinic. Thus, there is a low, but nonnegligible, risk (8%) of occult HSIL/AIN3 for subjects who test negative for hrHPV on anal swab.

Our cohort represents a high-risk population with known risk factors for anal HPV infection, including HIV infection, MSM, women with HPV disease at other genital sites, and a history of receptive anal sex. During the same study period, a total of 1,140 PLWHA had anorectal cytology and HPV contesting at our clinic. As anticipated, the majority tested positive for anal hrHPV (86%, unpublished data). In this substudy, we focused on the smaller subset of patients (14%) who tested negative for anal hrHPV.

In the context of cervical cancer screening, the Cobas® 4800 HPV test was reported to have a false negative rate of 0.7% in the multicenter, prospective ATHENA study [20]. The Hybrid Capture 2 (HC2) HPV DNA test (Digene Corp.) is another widely used HPV test in the clinical laboratory. The reported NPV for the HC2 test is also high in cervical cancer screening, ranging between 0.988 and 0.999 to 1.000 [21].

Anal squamous cell carcinoma is the major histologic type, and over 90% of cases are associated with HPV infection [22]. In anal cancer screening, HPV testing is known to have outstanding NPV and low false negativity. Two large studies confined to HIV-infected MSM reported NPV as high as 100% using HC2 Assay or Cobas [23, 24]. A meta-analysis calculated NPV to be 93.5% in a hypothetical population of 10,000 HIV-infected MSM [9]. Other groups reported NPV closer to ours, ranging from 81% to 94.5% using various HPV detection methods [25–27]. Studies pertaining to HIV-uninfected patients are very limited. Phanuphak et al. reported that the hrHPV DNA test has a NPV of 91% (95% CI 82.4–96.3) for HIV-uninfected patients [28].

Notwithstanding such optimal outcomes, histological diagnosis is still the gold standard and provides definitive evidence of disease. While HSIL generally entails persistent oncogenic HPV infection, HPV clearance can occur spontaneously and thereby account for a subset of negative HPV results. In the SUN study, anal HPV16 clearance and HPV18 clearance were 31% and 60% at 48 months in MSM, respectively [29]. Apart from HPV clearance, improper lab processing, and technical issues, we hypothesized three additional causes for the false negative cases based on the clinicopathological correlation.

The first cause relates to anal HSIL induced by HPV types that are not routinely screened for. Twelve of our high-grade lesions tested positive for HPV53, 67, 73, or 82 (patients 3, 8–14). Unlike the WHO-defined hrHPV types (i.e., IARC Group 1), these HPV types are currently designated as “possibly carcinogenic,” falling within the IARC Group 2B due to their low prevalence in cervical cancer (≤1% each) and the lack of biological data [30]. Although rare, anogenital precancers and cancers caused by “non-high-risk” HPV have been reported. In a study of 13,328 anogenital carcinomas, Guimerà et al. showed that ∼2% are caused by low-risk HPV 6, 11, 42, 44, or 70 [31]. Cornall et al. reported on two anal HSILs induced by low-risk HPV11 as verified by laser capture microdissection [32]. Though the molecular pathways remain unclear, our findings add to the existing literature on the possible carcinogenicity of HPV types currently considered “non-high-risk” and excluded from primary screening tests.

Secondly, patients with solitary and localized HSILs are likely to have negative HPV results via anal swab. Since anal swabbing is performed blindly, the large, corrugated anal canal surface is difficult to survey thoroughly; it is therefore easy to miss small, localized lesions. These factors could explain why seven of our false negative cases (patients 1–7) tested hrHPV negative on anal swabs but positive for hrHPV on biopsy specimens. All seven patients had only one localized high-grade lesion presumably missed by anal swabbing.

The third cause pertains to the methodology of HPV testing. Both the Cobas®4800 system and BMRT PCR assays are designed to target the HPV L1 gene, a region that can be lost during integration of viral DNA into host genomic DNA [33]. Consequently, L1-based PCR assays are limited by their inability to detect the integrated HPV. Patients 15–18 tested negative for hrHPV by both Cobas and BMRT assays; presumably, their oncogenic HPV was already present in an exclusively integrated form. Roberts et al. demonstrated that L1-based PCR failed to detect HPV16 in 3.9% of cervical HSILs due to viral integration [34]. In the context of anal lesions, 1.4% of HPV16 infections were reported to be in the integrated form [35]. Theoretically, L1-based tests would falsely categorize these patients as negative for HPV.

The strength of our study is that concomitant HPV testing and HRA-guided biopsy allowed for analysis of false negative HPV results. The study is limited in that random biopsies were not performed; as a result, disease burden may have been underestimated. Furthermore, as our cohort represents a population receiving effective antiretroviral therapy at a specialized HIV clinic, it may not be generalizable to patients in less rigorous clinical settings.

In conclusion, we found that anal HPV testing offered 92% NPV for PLWHA, in line with other comparable studies. There remains a low, but not negligible, risk (8%) of occult precancer. Our results should aid in the implementation of HPV testing in anal cancer screening programs. Future studies are needed to explore the optimal use of HPV testing whether alone, as cotesting with cytology or as a reflex test.

## Figures and Tables

**Table 1:** Clinicopathological characteristics for Cobas-negative individuals by HRA-guided biopsy result.

Characteristics	Total (*n* = 156)	HRA-guided biopsy			*p* value
		Benign (*n* = 56)	LSIL (*n* = 82)	HSIL (*n* = 18)	
Age (mean, range, yrs.)	49 (23–71)	48 (23–69)	49 (26–70)	49 (25–71)	0.73

Gender and sexual behavior					
MSM^*∗*^	129 (83)	46 (82)	67 (82)	16 (89)	0.10
HM^*∗∗*^	8 (5)	6 (11)	2 (2)	—	
Female	19 (12)	4 (7)	13 (16)	2 (11)	

Receptive anal sex					
Yes	145 (93)	48 (86)	79 (96)	18 (100)	0.03
No	11 (7)	8 (14)	3 (4)	—	

CD4+ T-cell count (cells/ml)					
<500	32 (21)	10 (19)	16 (20)	6 (35)	0.32
≥500	118 (79)	43 (81)	64 (80)	11 (65)	
Unknown	6	3	2	1	

HIV-1 viral load (copies/ml)					
<100	140 (90)	47 (84)	76 (93)	17 (94)	0.20
≥100	16 (10)	9 (16)	6 (7)	1 (6)	

Race/ethnicity					
Caucasian	33 (26)	6 (14)	22 (34)	5 (31)	0.10
African American	27 (22)	10 (23)	15 (23)	2 (13)	
Hispanic	47 (38)	20 (45)	23 (35)	4 (25)	
Other	18 (14)	8 (18)	5 (8)	5 (31)	
Unknown	31	12	17	2	

Smoking history					
Current	35 (22)	15 (27)	15 (18)	5 (28)	0.42
Former	46 (30)	14 (25)	29 (35)	3 (17)	
Never	75 (48)	27 (48)	38 (47)	10 (55)	

Anorectal cytology at the time of HRA					
Unsatisfactory	2 (1)	1 (2)	—	1 (5)	0.02
Benign	75 (48)	35 (62)	37 (45)	3 (17)	
ASCUS	62 (40)	18 (32)	33 (40)	11 (61)	
LSIL	13 (8)	1 (2)	10 (13)	2 (11)	
HSIL, ASC-H	4 (3)	1 (2)	2 (2)	1 (5)	

^∗^MSM: men who have sex with men; ^∗∗^HM: heterosexual men.

**Table 2:** HPV genotypes detected in high-grade lesions derived from Cobas-negative patients.

Cobas-negative patient	HPV type detected in high-grade lesion	
1	31	High risk
2	33	High risk
3^#^	35; 51	High risk
	73	2B^*∗*^
4	45	High risk
5	52	High risk
6	58	High risk
7	59	High risk
8	53	2B
9^#^	53	2B
	53	2B
10	67	2B
11	67	2B
12^#^	73	2B
	82	2B
13	82	2B
14^#^	67; 82	2B
	67; 82	2B
	67; 82	2B
15	Negative	
16	Negative	
17	Negative	
18	Negative	

^#^Patients 3, 9, 12, and 14 harbored multiple lesions. ^∗^2B: IARC category 2B.

## Data Availability

The data that support the findings of this study are available from the corresponding authors, upon reasonable request.

## References

[1] Siegel R. L., Miller K. D., Jemal A. (2019). Cancer statistics, 2019. *CA: A Cancer Journal for Clinicians*.

[2] Palefsky J. M., Holly E. A., Efirdc J. T. (2005). Anal intraepithelial neoplasia in the highly active antiretroviral therapy era among HIV-positive men who have sex with men. *AIDS*.

[3] Crum-Cianflone N. F., Hullsiek K. H., Marconi V. C. (2010). Anal cancers among HIV-infected persons: HAART is not slowing rising incidence. *AIDS*.

[4] D’Souza G., Wiley D. J., Li X. (2008). Incidence and epidemiology of anal cancer in the multicenter AIDS cohort study. *Journal of Acquired Immune Deficiency Syndromes*.

[5] Silverberg M. J., Lau B., Justice A. C. (2012). Risk of anal cancer in HIV-infected and HIV-uninfected individuals in North America. *Clinical Infectious Diseases*.

[6] Aberg J. A., Gallant J. E., Ghanem K. G., Emmanuel P., Zingman B. S., Horberg M. A. (2014). Primary care guidelines for the management of persons infected with HIV: 2013 update by the HIV medicine association of the infectious diseases society of America. *Clinical Infectious Diseases*.

[7] Curry S. J., Krist A. H., Owens D. K. (2018). Screening for cervical cancer. *JAMA*.

[8] Wentzensen N., Arbyn M., Berkhof J. (2017). Eurogin 2016 roadmap: how HPV knowledge is changing screening practice. *International Journal of Cancer*.

[9] Clarke M. A., Wentzensen N. (2018). Strategies for screening and early detection of anal cancers: a narrative and systematic review and meta-analysis of cytology, HPV testing, and other biomarkers. *Cancer Cytopathology*.

[10] Darragh T. M., Winkler B., Souers R. J., Laucirica R., Zhao C., Moriarty A. T. (2013). Room for improvement: initial experience with anal cytology: observations from the College of American pathologists interlaboratory comparison program in nongynecologic cytology. *Archives of Pathology & Laboratory Medicine*.

[11] Lin C., Franceschi S., Clifford G. M. (2018). Human papillomavirus types from infection to cancer in the anus, according to sex and HIV status: a systematic review and meta-analysis. *The Lancet Infectious Diseases*.

[12] Viciana P., Milanés-Guisado Y., Fontillón M. (2019). High-risk human papilloma virus testing improves diagnostic performance to predict moderate- to high-grade anal intraepithelial neoplasia in human immunodeficiency virus-infected men who have sex with men in low-to-absent cytological abnormalities. *Clinical Infectious Diseases*.

[13] Liu Y., Sigel K., Gaisa M. M. (2018). Human papillomavirus genotypes predict progression of anal low-grade squamous intraepithelial lesions. *The Journal of Infectious Diseases*.

[14] Umberger R. A., Hatfield L. A., Speck P. M. (2017). Understanding negative predictive value of diagnostic tests used in clinical practice. *Dimensions of Critical Care Nursing*.

[15] Bean S. M., Chhieng D. C. (2010). Anal-rectal cytology: a review. *Diagnostic Cytopathology*.

[16] Sun Z., Zhang R., Liu Z. (2015). Development of a fluorescence-based multiplex genotyping method for simultaneous determination of human papillomavirus infections and viral loads. *BMC Cancer*.

[17] Jay N., Berry M. J., Hogeboom C. J., Holly E. A., Darragh T. M., Palefsky J. M. (1997). Colposcopic appearance of anal squamous intraepithelial lesions. *Diseases of the Colon & Rectum*.

[18] Darragh T. M., Colgan T. J., Thomas Cox J. (2013). The lower anogenital squamous terminology standardization project for HPV-associated lesions. *International Journal of Gynecological Pathology*.

[19] Schiffman M., Clifford G., Buonaguro F. M. (2009). Classification of weakly carcinogenic human papillomavirus types: addressing the limits of epidemiology at the borderline. *Infectious Agents Cancer*.

[20] Stoler M. H., Wright T. C., Sharma A., Apple R., Gutekunst K., Wright T. L. (2011). High-risk human papillomavirus testing in women with ASC-US cytology. *American Journal of Clinical Pathology*.

[21] Burd E. M. (2016). Human papillomavirus laboratory testing: the changing paradigm. *Clinical Microbiology Reviews*.

[22] Saraiya M., Unger E. R., Thompson T. D. (2015). US assessment of HPV types in cancers: implications for current and 9-valent HPV vaccines. *JNCI: Journal of the National Cancer Institute*.

[23] Salit I. E., Lytwyn A., Raboud J. (2010). The role of cytology (Pap tests) and human papillomavirus testing in anal cancer screening. *AIDS*.

[24] Wentzensen N., Follansbee S., Borgonovo S. (2012). Human papillomavirus genotyping, human papillomavirus mRNA expression, and p16/Ki-67 cytology to detect anal cancer precursors in HIV-infected MSM. *AIDS*.

[25] Burgos J., Hernández-Losa J., Landolfi S. (2017). The role of oncogenic human papillomavirus determination for diagnosis of high-grade anal intraepithelial neoplasia in HIV-infected MSM. *AIDS*.

[26] Goldstone S. E., Lowe B., Rothmann T., Nazarenko I. (2012). Evaluation of the hybrid capture 2 assay for detecting anal high-grade dysplasia. *International Journal of Cancer*.

[27] Hidalgo-Tenorio C., Rivero-Rodriguez M., Gil-Anguita C. (2015). The role of polymerase chain reaction of high-risk human papilloma virus in the screening of high-grade squamous intraepithelial lesions in the anal mucosa of human immunodeficiency virus-positive males having sex with males. *PLoS One*.

[28] Phanuphak N., Teeratakulpisarn N., Keelawat S. (2013). Use of human papillomavirus DNA, E6/E7 mRNA, and p16 immunocytochemistry to detect and predict anal high-grade squamous intraepithelial lesions in HIV-positive and HIV-negative men who have sex with men. *PLoS One*.

[29] Patel P., Bush T., Kojic E. M. (2018). Prevalence, incidence, and clearance of anal high-risk human papillomavirus infection among HIV-infected men in the SUN study. *The Journal of Infectious Diseases*.

[30] WHO (2007). IARC monographs on the evaluation of carcinogenic risks to humans. *Human Papillomaviruses*.

[31] Guimerà N., Lloveras B., Lindeman J. (2013). The occasional role of low-risk human papillomaviruses 6, 11, 42, 44, and 70 in anogenital carcinoma defined by laser capture microdissection/PCR methodology. *The American Journal of Surgical Pathology*.

[32] Cornall A. M., Roberts J. M., Garland S. M., Hillman R. J., Grulich A. E., Tabrizi S. N. (2013). Anal and perianal squamous carcinomas and high-grade intraepithelial lesions exclusively associated with “low-risk” HPV genotypes 6 and 11. *International Journal of Cancer*.

[33] Tjalma W. A. A., Depuydt C. E. (2013). Cervical cancer screening: which HPV test should be used-L1 or E6/E7?. *European Journal of Obstetrics & Gynecology and Reproductive Biology*.

[34] Roberts C. C., Tadesse A. S., Sands J. (2006). Detection of HPV in Norwegian cervical biopsy specimens with type-specific PCR and reverse line blot assays. *Journal of Clinical Virology*.

[35] Alvarez J., de Pokomandy A., Rouleau D. (2010). Episomal and integrated human papillomavirus type 16 loads and anal intraepithelial neoplasia in HIV-seropositive men. *AIDS*.

